# The New Basis of Nursing Education

**Published:** 1921-02-12

**Authors:** 


### THE MATRONS' AND SISTERS' DEPARTMENT.

### THE NEW BASIS OF NURSING EDUCATION.

GENERAL disappointment is felt at the long delay of the Ministry of Health in dealing with the matters referred by the General Nursing Council. The draft Rules for Registration were submitted last September. The resolution relating to the vexed question of nurses' hours of employment was forwarded in December. Neither matter has yet been replied to, and the General Nursing Council has decided not to meet again until an official reply has been received from the Minister on matters which have been submited to him. In a matter of such deep importance to nurses and to the public no one would wish to see rules hastily adopted. But the lawyers and other wise heads have now had over four months to reflect upon them, and should surely be within measurable distance of detecting every possible objection.

Meantime, the education and examination committee has reached certain definite conclusions. The first relates to the drawing up of a syllabus of nursing education to be circulated to all training-schools for nurses. The preparation of this syllabus is one of the weightiest tasks which the Council will have to perform, and they are fortunate in having the advice and long experience of Miss Lloyd Still in dealing with it. Many years ago when this able matron was directing the nursing at the Brompton Hospital for Consumption she turned her attention to the need for codifying the elements of nursing education. During her term of office at the Middlesex Hospital she enlarged her methods, which have been subsequently perfected at St. Thomas's, and we know of no more admirable system for ensuring a knowledge of every detail in a nurse's work than the one thus at length brought into being. If something on these lines be adopted by the Council, there cannot be two opinions about its probable effect in raising the standard of nursing proficiency all over the country.

The Council took an important step on February 2 in fixing dates for the first examinations under the Act. The first four State examinations are to be voluntary, that is to say, that candidates for registration may enter for them or not as they desire. These four will be held in July and October 1923, January and April 1924. After the last of this series no candidate will be registered without passing the State examination. The first examination compulsory on all candidates for registration is to take place in July 1924, after which quarterly examinations will take place in fourteen centres.

Every effort is to be made to bring to the notice of nurses and their training-schools the decisions of the General Nursing Council. We are certain that a very energetic forward policy must be adopted to this end. We welcome the decision of the Council to draw up a Bulletin, to be widely circulated, showing what is being done by the Council, but it will require very wise handling if it is to carry weight and bring in adhesion to the schemes for higher education. There is no doubt at all that the spoken word has more effect on many nurses than the most carefully thought-out manifesto, but the decision to pay the expenses of members of the Council to visit and address nurses in various areas is in our judgment one of doubtful expediency. Such action might help to accentuate the fact that the General Nursing Council is composed of elements not entirely fused. What some members said might be conceivably resented by some others, and the way would be open to expose a lack of cohesion, possibly of harmony. It seems to us that the proper course for the Council to pursue in promoting the propaganda necessary for the success of their work is to engage an organising secretary to represent the views of the Council as a whole and bring them to the notice of nurses in every part of the country.

The recommendation (which was adopted) to hold an informal conference of matrons and teachers of nurses on the subject of future training is excellent. The Council should never lose sight of the fact that registration will be purely voluntary. They cater for a profession which can reject their proposals if it decides to do so. Hence the imperative need for enlisting the sympathy of every one interested in the education of nurses and of bringing all sections of nursing in one way or another into the curriculum.

